# The influence of the psychological contract on employee engagement in a South African bank

**DOI:** 10.3389/fpsyg.2022.958127

**Published:** 2022-08-02

**Authors:** Dzunani A. Ngobeni, Musawenkosi D. Saurombe, Renjini M. Joseph

**Affiliations:** Department of Industrial Psychology and People Management, College of Business and Economics, University of Johannesburg, Johannesburg, South Africa

**Keywords:** psychological contract, employee engagement, banking, line manager, COVID-19 pandemic

## Abstract

The workplace is characterised by numerous contracts of agreement that an employee and employer must sign to formalise their employment relationship. The informal agreement, known as the psychological contract, is often overlooked, although it is pivotal in determining the engagement of employees in an organisation. This study aimed to probe the perceptions line managers have of the influence of the psychological contract on employee engagement in a South African bank with a particular focus on how the integration of technologies from the Fourth Industrial Revolution may have impacted the workplace in the banking sector. The study was carried out using a qualitative research approach. A purposive random sampling strategy was used to select participants who were interviewed using semi-structured, one-on-one interviews. The data collected were analysed using thematic analysis, and verbatim quotes were used to support emergent themes. The findings of the study revealed that continuous change in the world, exacerbated by the COVID-19 pandemic, influences employee expectations. Thus, organisations must be able to quickly adapt and adjust their talent attraction and retention mechanisms. Talent management, the nature of the business, structure and operations, the nature of the work environment, and emotional needs are the themes that emerged from the study. With the constant change in the world of work, including industry disruptions continually imposed by the 4IR and other factors, employees’ expectations are ever-changing. Thus, organisations must keep adapting to attract and retain talent. This study adds value by addressing various aspects aligned with competitively adjusting to the current and future world of work.

## Introduction

The workplace is characterised by numerous contracts between an employee and employer that formalise their employment relationship. This includes the employment contract and other formal documents such as non-disclosure agreements. However, there is an informal agreement that is often overlooked, and is pivotal in determining the engagement of employees in an organisation. Such an informal agreement is known as the psychological contract (Naidoo et al., 2019). The psychological contract is important to enhance the employer–employee relationship. The psychological contract represents the mutual beliefs, perceptions, expectations and informal obligations between an employer and an employee (Saurombe and Barkhuizen, 2020). It entails the unwritten rules that govern the reciprocal social dynamics underlying the relationship between the two parties and defines the tasks to be completed in practical terms (Gordon, 2020). While the psychological contract is informal, the literature suggests that it should not be overlooked as it sets the scene for the employer-employee relationship (Bussin, 2021; Holland and Scullion, 2021; Mmamel et al., 2021).

Employee engagement refers to the extent to which an employee feels passionate about their job, is committed to the organisation, and puts effort into his work (Saad et al., 2021). Engaged employees exude self-efficacy, a high level of optimism and resilience, and can control and achieve success in their career by having self-esteem (Koveshnikov et al., 2020). Line managers are therefore encouraged to assess employees’ perceptions of the psychological contract. This study explores the perceptions of line managers about how obligations and expectations of employees and employers in the form of the psychological contract can influence employee engagement in a South African bank, specifically considering how the integration of technologies from the Fourth Industrial Revolution (4IR) may have impacted the banking workplace. The objectives of the study were to explore the perceptions that line managers have of employee expectations and employee engagement, and to explore the perceptions of how the psychological contract can influence employee engagement in a South African bank.

The study looks at the financial services sector, with a focus on the banking industry in South Africa. The study further seeks to understand how the integration of technologies from the 4IR in the workplace has changed the nature of the banking sector and how it operates, and its subsequent effect, on the psychological contract. Banks in South Africa must rethink strategies for employee attraction, development and retention as a result of the 4IR (Business Tech, 2020; PWC, 2020). Employees’ expectations are rapidly shifting due to changing nature of work as a result of technological changes and advancements (Sutherland, 2020; Prakash et al., 2021). The authors’ motivation to carry out this study in the South African banking sector emanates from the minimal research available on the contemporary context of the influence of psychological contracts on employee engagement given the current rapidly evolving workplace environment.

## Literature review

### Cognitive dissonance theory

Leon Festinger first coined the theory of cognitive dissonance at the beginning of the 1950s. The theory suggests that there can be inconsistencies among cognitions such as knowledge, opinions, beliefs and behaviour, thus generating an uncomfortable motivating feeling, also known as cognitive dissonance (Festinger, 1957). Whenever an employer creates an expectation in an employee in exchange for the employee’s contribution and does not meet the expectation, this will limit the motivation of the employee to produce the desired work outcome (Alcover et al., 2017). According to the cognitive dissonance theory, when an employee perceives a breach of the psychological contract or the employer fails to fulfil their promised obligations, the employee may view these unfulfilled expectations as a form of wrongdoing on the part of the employer (van Gilst et al., 2020). This may consequently leave the employee experiencing cognitive dissonance, therefore feeling dissatisfied with the employment relationship.

Denial and contradiction are identified as the root causes of a breach of the psychological contract (Li and Chen, 2018). Denial occurs when an organisation creates an expectation but fails to fulfil it (Li and Chen, 2018). Contradiction occurs when there are differences in the understanding of whether an expectation exists or not (Abbas and Al Hasnawia, 2020). The resulting cognitive dissonance could result in lowered engagement, reduced performance, loyalty and job satisfaction. According to Dwivedi et al. (2018), understanding cognitive dissonance is important and helps line managers address the misaligned beliefs that result in dissatisfied attitudes, thus streamlining the expected behaviour of employees within an organisation.

Line managers can use the understanding and prevention of cognitive dissonance to encourage successful change, or otherwise impede it (Holley, 2021). Understanding the possibility and impact of the manifestation of cognitive dissonance in the workplace is also critical to ensuring and maintaining employee engagement (Perrigo, 2021). This theory postulates that when a person’s behaviour is inconsistent with their thoughts and beliefs, underlying psychological tension builds up (Saurombe and Barkhuizen, 2020). This psychological tension can motivate an individual to change their attitude, thus producing consistency between their thoughts and behaviours.

### The nature of the psychological contract

The concept of the psychological contract was first introduced by Argyris (1960). On analysing interviews conducted between employees and supervisors in two factories, Argyris (1960) found the psychological contract to be an implicit understanding between the group of employees and their foreman. Argyris (1960) further argued that the relationship could develop to allow employees to exhibit higher productivity and fewer grievances in exchange for acceptable wages and job security. The defining characteristics of the first explicit conceptualisation of the psychological contract was an exchange of tangible, specific and economic resources agreed upon by the two parties that allowed for the fulfilment of the needs (Argyris, 1960).

Tomprou et al. (2015) note that, unlike a formal contract, a psychological contract represents the intangible mutual beliefs, perceptions and informal obligations between an employer and an employee. Psychological contracts are informally established as an implicit agreement between the employee and the employer on the roles and activities that each party is going to perform without specifically elaborating on the details (Swanepoel and Saurombe, 2022). It is predominantly subjective and dependent on what the employee and the employer believe is acceptable or not (Rousseau et al., 2018). Although the psychological contracts are informally established, the employer and employee should explicitly live by the expectations (Prakash et al., 2021).

Holland and Scullion (2021) are of the opinion that the psychological contract is important to an organisation as it provides the framework for understanding the nature of employment relationships and the impact that they can have on the economic outcomes of the organisation.

The concept of the psychological contract has attracted attention mostly for two reasons; first, it is a way of understanding and managing the attitude and behaviours of employees within a company (Agarwal et al., 2021); and secondly, it helps us understand how the relationship between employee and employer evolves (Griep and Vantilborgh, 2018). A breach of a psychological contract occurs when either the employee or employer feels that the other party has not fulfilled a perceived expectation (Gulzar et al., 2021). A breach of this contract can lead to either party feeling psychologically and emotionally aggrieved, therefore leading to a loss of loyalty, motivation, and overall, duty underperformance (Gulzar et al., 2021).

### The psychological contract in the banking sector

The banking sector is changing at a rapid pace and is becoming more competitive than ever before (Rasool et al., 2021). The changes in the banking environment have brought a shift in the nature of the employer-employee relationship. Traditionally, the psychological contract has been geared to aspects such as trust, respect and loyalty between the employer and the employee (Swanepoel and Saurombe, 2022). These relational aspects are still important, however, the recent insecure and changing work environment has brought a shift so that we are starting to see transactional exchange becoming more prevalent (Braganza et al., 2021). Advanced technology is becoming pervasive and competition in the banking sector is increasing (Rasool et al., 2021). For banks to keep performing at their best at all times, they need a committed and motivated workforce who give their very best to achieve their strategic objectives. Employees are increasingly seeking self-actualisation more than the monetary rewards that banks are willing to offer in exchange for their skills. Self-actualisation is the highest human need according to Maslow’s hierarchy (Barnes, 2021). Banks need more skilled employees at every level of the organisation as a result of new markets, tighter competition and the emergence of technologies (Braganza et al., 2021).

### The psychological contract and employee engagement

According to Naidoo et al. (2019), employee engagement refers to the extent to which an employee’s mind and heart are captured by an organisation, thus helping organisations to perform better. Energy, involvement, and positive interaction are regarded as employee engagement characteristics (Naidoo et al., 2019). In other words, employee engagement has the power to drive the performance of the organisation. According to Agarwal and Sajid (2017), engaged employees understand their position and purpose in the organisation, which allows them to become motivated and driven enough to perform tasks allocated to them with passion. These employees are identified by Agarwal and Sajid (2017) as the engine of the organisation and they support the organisation to achieve its mission and effectively execute its strategy, thus generating business results. According to Naidoo et al. (2019), engagement is a multidimensional construct. The emotional and cognitive dimensions are briefly explained below. The fulfilment of the psychological contract increases employee engagement, and morale for the job and in the workplace, and decreases turnover intentions (Swanepoel and Saurombe, 2022).

#### Emotional engagement

Emotional engagement entails connecting with colleagues and managers in a meaningful way. Employees contribute positively to organisational effectiveness when they feel that their managers are interested in their development (Naidoo et al., 2019; Jarrar, 2022). Employees put in more effort, and work and give a better performance when they feel that these make a difference in the organisation (Chanana, 2021). In the study that Sandhu and Sharma (2022) conducted, they found that emotional satisfaction, perceived fairness, personal development, and culture, clear communication, and compensation are the drivers of employee engagement. Similarly, the study conducted by Saad et al. (2021) found that hiring and selection, training and development, and job security are important in enhancing employee engagement levels. Thus, line managers need to invest time and effort in the emotional engagement and the development of their employees (Shirin and Kleyn, 2017).

#### Cognitive engagement

The dimension of cognitive engagement acknowledges when an employee is aware of their mission, role in, and contribution to the organisation (Abarantyne et al., 2019). Employees are more likely to excel when they understand their purpose in the organisation and are provided with opportunities to grow (Saurombe et al., 2017). Engaged employees are those who can connect with others in the work environment and who know precisely what is expected of them (Abarantyne et al., 2019; Naidoo et al., 2019). Line managers should continuously reaffirm the employee’s purpose and expectations.

For employees to be personally engaged, they must be more engaged in both dimensions (Dash, 2021). Organisations have to meet the expectations created to ensure that employees are engaged with the organisation’s objectives (Dash, 2021). When employees’ expectations are not met, this can result in a breach of the psychological contract, as such a breach is defined as an organisation’s failure to meet the created expectations (Gulzar et al., 2021). Breach of the psychological contract may negatively affect the employee’s attitudes and behaviours toward an organisation, leading to a loss of confidence, respect, and possible negative emotional responses (Abbas and Al Hasnawia, 2020). Psychological contracts are integral to helping navigate workplace dynamics because these informal agreements frame all formal transactions (Swanepoel and Saurombe, 2022). Line managers must be mindful of psychological contracts when selecting, training, retaining and promoting employees. Trust is an important aspect of the social relationship between an employer and an employee (Gulzar et al., 2021).

### The psychological contract in the context of the Fourth Industrial Revolution

Organisational and employee expectations are continuing to evolve at a fast pace as a result of the 4IR. Recently, in light of the COVID-19 pandemic, South African organisations have not only continued discussions on 4IR transformations but accelerated their implementation (Kwon and Jang, 2021). 4IR (digital technology) includes the Internet of Things, big data, cloud computing, artificial intelligence, as well as machine learning (Kwon and Jang, 2021). The accelerated technological revolution not only play a vital role in bolstering organisational productivity and competitive advantage but the creation of wealth (Aderibigbe, 2021). Digitalisation has an impact on human capital, resulting in a shortage of some and redundancy in other skills as a result of some jobs becoming automated (Magagula et al., 2020; Kwon and Jang, 2021). The nature of work is changing as a result of digitalisation, and employees have to adjust. Organisations are required to adapt and adjust their human capital practices based on the changes brought by digitalisation to meet the evolving needs and expectations of the labour market (Aderibigbe, 2021; Harris, 2021).

Some of the human resource (HR) strategies that organisations have to adjust include, but are not limited to, talent attraction needed for the 4IR, and retaining and developing employees (Dhanpat, 2021; Hewapathirana and Almasri, 2021). Additionally, the development and introduction of new organisational and work models can help address the said changes. These practices mean that employees’ psychological contracts should be renewed or adjusted (Dhanpat et al., 2019). According to the study conducted by Chinyamurindi (2021), while the acceleration of the 4IR does pose threats and affect the psychological contract, there is a greater need for HR to be a strategic business partner, increasing the need for the psychological contract. Malik et al. (2022) add that the use of digital technology can be cost-effective and enhance the overall employment experience of employees and thereby resulting in employee commitment, satisfaction, and reduced turnover.

In reviewing the literature, the nature of the psychological contract and related theories were outlined. The psychological contract in the banking sector and within the context of 4IR were discussed. Specifically, the link between the psychological contract and employee engagement was probed. This review of the literature highlights the importance of exploring the line managers’ perceptions of the influence of the psychological contract on employee engagement, specifically in the banking context, taking into consideration 4IR. The methodology adopted to address the research objectives are discussed next.

## Methodology

### Research approach and philosophy

This study followed a qualitative approach, as the targeted participants (middle managers) form a small population at the selected bank, which disqualified the use of a quantitative approach. Further, the target sample was chosen since they are close custodians of the psychological contract, while simultaneously being close influencers of employee engagement through the performance review process (van Elst and Meurs, 2016). What is more, the analysis of in-depth insights (qualitative) was more critical to this research than numbers (quantitative). Braun and Clarke (2021a) argue that the qualitative research design seeks to understand people’s beliefs, behaviours, experiences, and interactions. Non-numerical data are generated with this method, and it further helps to gain increased insight and understanding across disciplines (Bogner et al., 2018). The chosen approach was interpretative and inductive and provided insights into how participants see their reality as well as how they understand or see their experiences.

This study departed from an interpretive view, which is defined as a paradigm that considers or accepts that true knowledge can be obtained from interpreting people and understanding concepts from exploring surroundings (Saunders et al., 2016). Interpretivism is based on the consideration that humans can offer depth of meaning and can effectively attach those meanings to variables and factors (Alharahsheh and Pius, 2020). Saunders et al. (2016) describe ontology as dealing with ideas relating to the existence of relationships between people, society and the world. The ontology was to explore and understand how participants are affected by the psychological contract as their truth. Saunders et al. (2016) further add that ontology is based on social phenomena and how meanings are constructed continually by social factors. In this study, the main author interacted with the participants to discover and understand their experiences by probing further for insight into their ability to create meaning in the world of work. Saunders et al. (2016) argue that roles, narratives, stories, and personal interpretations that are part of the study can be acknowledged as epistemology. The epistemological stance was to acknowledge the role that narratives and personal interpretations play in the psychological contract.

Semi-structured interviews can provide insight into the views and perceptions of participants. It is also important to highlight that the main author was exposed to subjectivism and committed to being objective during the interviews. The axiology acknowledged the subjective interpretation of the responses in terms of the values and ethics that exist. Further, it was important to respect the main author’s values and those of the participants during the data collection process, and it is acknowledged that different values did exist, leading to possible subjectivity on the part of either party (Saunders et al., 2016).

### Research participants and sampling

The participants in this study consisted of 13 middle managers as they are the first level of managers who interact with employees on matters of employee engagement. They are often also responsible for conducting employee performance conversations and reviews. The research participants were selected from a South African bank. The inclusion criteria used to select participants were that managers had to have at least 5 years of managerial working experience, had to reside in Johannesburg, South Africa, and could be of any ethnic and gender group. Further, the participants were approached based on meeting the minimum inclusion criteria as mentioned in the preceding statement.

The authors selected the purposive sampling strategy for this study. According to Saunders et al. (2016), purposive sampling provides the researcher with a subjective criterion to select the sample of participants. Gichuru (2021) highlights that cost-efficiency and time saving are some of the advantages associated with this strategy. One of the disadvantages of this sampling strategy is the limited generalisability of the findings (Andrade, 2021). The rationale for choosing this strategy was limited time, budgetary constraints and the specific need for line manager participants to reach the study objectives. The population targeted for this study consisted of employees working in a South African bank in Gauteng. The sample size of the study was 13 participants. Sim et al. (2018) suggest that good sample size for a qualitative study would be between 6 and 15 participants. It is important to mention that in the eighth interview, no new themes emerged. The main author, therefore, did not continue exploring the subject beyond the thirteenth interview as data saturation was reached. This is in line with the study conducted by Braun and Clarke (2021b), which found that 94% of the most frequent themes emerge in the first 6 interviews and 97% by 12 interviews, implying that the sample size of this study is satisfactory to ensure valid findings.

### Data collection

A research interview schedule or guide with semi-structured interview questions designed to garner profound insight into the research topic was used. The interview guide included questions about the line managers’ understanding of employee expectations, the extent to which these expectations are met contractually, the role line managers play in meeting such expectations, how they understand employee engagement, the role that they play in ensuring that their employees are engaged, and how they understand the COVID-19 pandemic affected the psychological contract and employee engagement (Soares and Sidun, 2021). For the main researcher and participant to discuss some questions in more detail, the questions that were asked were open-ended in nature. Semi-structured interviews allowed more information to be collected by allowing the researcher to probe for further elaboration on answers provided by the chosen sample of employees (Foley et al., 2021). The prospective participants were contacted via email and/or telephone to set up one-on-one interview meetings. The interviews were conducted face-to-face by the main author as was permitted by the South African national COVID-19 lockdown regulations and restrictions at the time of data collection. The interviews, which were all conducted in English, were audio-recorded with the consent of the participants and used for this study. The recorded interviews were stored safely while adhering to the anonymity and protection of personal information. To achieve and verify the quality of the answers, the data collected was measured for validity and reliability by means of triangulation (Saunders et al., 2016).

### Data analysis

The main author began the process of transcribing the audio recordings after the data collection process. The authors grouped the transcribed data into codes and began the thematic analysis thereafter. The process involved thematic analysis and an inductive approach to coding where themes were allowed to organically emerge. The authors manually went through the transcripts, using different colours to highlight different codes and ultimately, categorised themes (Caulfield, 2019). The inductive approach to coding gave rise to insights beyond the theoretical underpinnings of this study, contributing to its significance and value-add (Linneberg and Korsgaard, 2019; Eger and Hjerm, 2022). Braun and Clarke (2021a) define thematic analysis as a method used for systematically identifying, organising and offering insights into patterns about meaning across a set of data. Thematic analysis with an inductive approach allowed the authors to conclude collective or shared meanings and experiences by focusing on meaning across a set of data. The authors first reviewed the data to discover other domains by using coding that is open and selective. Once the domains were discovered, open codes were broken down from the derived data into groups to allow the identification of relationships among the codes (Jowsey et al., 2021). Data were assessed using analytical and logical reasoning. Once the data had been coded, it was arranged into common emerging themes and categories. This process ensured order, structure and interpretation of collected data (de Farias et al., 2021).

Braun and Clarke (2021a) propose six steps to thematically analyse data. First, the main author became familiarised with the data by listening to the recordings and making additional notes. Second, initial codes were generated/formulated, after which the authors met to discuss common codes, answering the research questions by using an analysis matrix. Third, the authors explored data themes by reviewing and grouping the codes according to their similarities. Once grouped, the authors brainstormed possible themes that would have emerged. Fourth, the themes were reviewed, re-examined, and regrouped by the authors. The themes were then labelled and defined accordingly and aligned with the research questions. Lastly, the authors produced a written report and supported the findings with verbatim quotes from the interview responses.

### Ethical considerations

Ethics clearance was sought from and granted by the Research Ethics Committee of the College of Business and Economics at the University of Johannesburg with Ethics Clearance Reference Number: IPPM-2021-587(M). The study was planned with the necessary thoroughness to eliminate any information that may have been misleading, and the study conformed to the expected ethics considerations. The dignity, confidentiality and welfare of the participants and anyone who had been impacted by the study were protected and guaranteed. Clark-Kazak (2017) outlines the following ethical considerations, which were observed during this study as well.

#### Equity

It was ensured that the relationship with interviewees was an equitable one by being aware of any dynamics of power that may have arisen, thus guarding against the same.

#### Right to self-determination

The participants’ dignity was upheld throughout the research process by ensuring that the participants understood the voluntary nature of participation in the study, as well as their right to refuse to participate in the study without repercussions. Full information about the study was disclosed to participants.

#### Voluntary participation

Participation in the study was voluntary and without coercion. Participants were allowed to withdraw from the research process at any time if they felt uncomfortable or unable to continue.

#### Informed consent

Participants were requested to formally consent to partake in the study by signing a consent form/letter. All participants included in the study signed the consent forms.

#### Protection of participants

Participants were protected from any physical and or mental harm. Pseudo names (e.g., participant 1, participant 2, and so forth) were used to replace the “true” identity of participants.

#### Confidentiality

Participants were reassured that all personal information will be kept confidential. Participants’ true identities were protected by using pseudo names. Identifiable information about the participants was not and will not be disclosed. The participants were reassured that the information provided during the data collection process will not be deliberately disclosed to others except to the University of Johannesburg and its associated stakeholders linked to this study while protecting the participants’ true identities.

## Findings

The purpose of this study was to obtain insights from line managers on the influence of the psychological contract on employee engagement through the employment of structured interviews. A thematic analysis using an inductive coding approach was employed to analyse the data that had been collected, and 22 codes emerged. The emergent codes were further collapsed into four main themes and 20 sub-themes, as presented in Table 1.

**TABLE 1:** Categories of main and sub-themes.

Main theme	Sub-themes
Talent management	Managerial support, and career growth empowerment
	Performance management
	Remuneration and employee benefits Rewards and recognition Talent recruitment and retention Quality of outputs Meaningful work and job satisfaction
Business nature, structure and operations	Business purpose and expectations
	Work flexibility/autonomy
	Teamwork
	Characteristics of the institutional psychological contract
	The role of technology
	Leadership
	Organisational culture and change management
Nature of work environment	Occupational health and safety Respect, fairness and transparency Job security
Emotional needs	Humanity/sense of belonging
	Emotional support and morale Loss

### Main theme 1: Talent management

Talent management was the most frequently emerging theme found in the study. According to the line managers, employees expect managerial support and to be empowered to carry out their responsibilities. Performance management, remuneration and employee benefits should be fair. Employees also expect to be rewarded and recognised for a job well done and for the quality of outputs. Talent recruitment and retention strategies are expected to be applied consistently. Lastly, employees expect to be given meaningful and purposeful work, leading to their perceived job satisfaction.

#### Sub-theme: Managerial support, empowerment, and career growth

This sub-theme occurred most frequently within the broader theme of talent management. Many line managers indicated that employees expect a supportive work environment from their employment experience and to be empowered in the execution of their responsibilities. Below are some of the responses that support these findings:

*“I like to have an empowered team that goes and does things but within the safeguards of the guidance that I provide, to make sure that if we are heading in that direction, we’re heading in the right direction. So, my key strategy is still around that ongoing staff engagement process, so I’m always engaging them. I don’t know how to put it differently, but it’s, it’s around empowering them.”* (Participant 10, female, African, 11+ years of work experience)

Other participants added:

*“It’s the availability of the lead* [ership] *as a line leader to them* [and] s*tretching* [them in terms of] *what they need to deliver. You will be able to also ensure that they are thinking beyond just the scope of their work so that there is some kind of stretch within what they can build themselves from a career perspective.”* (Participant 4, female, coloured, 11+ years of work experience)

*“People expect empowerment.”* (Participant 11, female, Indian, 20+ years of work experience)

Leadership visibility was another theme that came from the participants. Participants believe that some of the employees’ expectations are for their line managers to be available, present, supportive and provide guidance. Some of the comments from the participants are included below:

*“I always encourage that if you have a problem, and you’ve consulted your leader, and you see it’s not working, and you’ve escalated, and you follow the chain of command, but you see that you’re just hitting a brick wall, I have an open-door policy that says come and talk to me, I have no issues with that.”* (Participant 11, female, Indian, 20+ years of work experience)

*“…Not only financially, emotionally, but also psychologically, people are going through a lot and they need support. In that regard, they’re looking for avenues. You don’t have to solve all of it, but can you point them in the right direction…”* (Participant 13, male, African, 11+ years of work experience)

From the line managers’ perspective, growth through empowerment and learning was important to employees. Most of the participants believed, based on their continuous engagement with their teams, that the employees in the bank expect career growth/progression through continuous learning, being empowered, and ultimately earning a higher salary, as encapsulated in the following views:

*“I think there are two things. One is career progression, which is linked to growth…”* (Participant 3, male, African, 16+ years of work experience)

*“I do believe, especially with the younger generations that we have now, they want to see quick changes, they want to see growth, quick growth moves, they want to see, I’ve learned this now and I can move on.”* (Participant 11, female, Indian, 20+ years of work experience)

*“…everybody talks about the Fourth Industrial Revolution but there is an expectation from employees to organisations where even amid the potential that AI* [Artificial Intelligence] *and robotics can take my job, how do you help me to remain secure in my job tomorrow? It is still about security. It is about the learning interventions that organisations can provide. Is there may be a bursary that can afford me to study something different so that I can make myself relevant in the world of work that is constantly changing before my eyes?”* (Participant 13, male, African, 11+ years of work experience)

Most line managers believed that employees expect career advancement opportunities from their employment experience, as supported by the below quotes:

*“They want to learn, to grow, to be able to further their path in their career.”* (Participant 2, female, African, 16+ years of work experience)

*“I think there are two things, one is career progression…”* (Participant 3, male, African, 16+ years of work experience)

*“…and then growth and understanding the growth path in terms of where to from here almost would be important for them as well.”* (Participant 9, female, coloured, 16+ years of work experience)

#### Sub-theme: Performance management

The theme of performance management was the second most frequently occurring sub-theme under the broader theme of talent management. The performance management process in the workplace is critical because it helps organisations and line managers identify if employees are meeting the set targets toward achieving the bank’s objectives. With the COVID-19 pandemic that has impacted the world of work, line managers have had to be innovative in the way in which they manage employees during the current pandemic and will continue to reshape the way performance management processes are handled post-COVID-19 pandemic, given the expected hybrid working model by employees. Below are some of the comments from line managers supporting this:

*“I think COVID is going to be around for a while because we haven’t reached that critical mass. I think the thing is around the hybrid working model will still be there and how do we really implement it. How do we contract* [performance management] *with individuals around their work deliverables?”* (Participant 4, female, coloured, 11+ years of work experience)

*“…you’re supposed to engage with a performance feedback session annually, which is your mature performance, your hearing performance. So those are the two official contractual means in place, but we also have, I think, over and above, regular engagement with the employees, so they know exactly what is expected.”* (Participant 8, female, White, 20+ years of work experience)

Another participant added:

*“You would have your performance management process where you would look at the growth of the individual you contract, where they want to go to and achieve in terms of their career… where gaps are identified then you and* [that] *individual have a responsibility to work towards closing those gaps.”* (Participant 4, female, coloured, 11+ years of work experience)

#### Sub-theme: Remuneration and employee benefits

Employees expect to be remunerated accordingly and fairly, making them able to fend for their families and have a quality of life. Several participants mentioned that employees’ biggest expectation is to earn fair and reasonable remuneration. Below are some of the comments that support this:

*“They also expect fair and reasonable remuneration…”* (Participant 6, female, white, 16+ years of work experience)

*“…obviously, salary benefit, you know, that that would also be critical because I think that’s the reason why they work, for the salaries and the benefits and being able to feed their families.”* (Participant 8, female, white, 20+ years of work experience)

Other participants implied that while other factors are important, it mostly comes down to equitable and lucrative compensation, as indicated by the following perspectives:

*“It’s all very encompassing. It can’t just be one thing. So, when I come in as a human being, I’ve got my own needs and for those needs to be met, we meet them from a remuneration perspective…”* (Participant 5, female, African, 16+ years of work experience)

*“So, the first one is to be paid…”* (Participant 1, female, white, 16+ years of work experience)

#### Sub-theme: Rewards and recognition

Line managers believed that employees expect rewards and recognition by celebrating successes and being remunerated accordingly in line with the quality of outputs and efforts, as supported by the following responses:

*“…if it is required, to havex more hands-on involvement, as well, but also celebrating successes.”* (Participant 8, female, white, 20+ years of work experience)

*“They* [employees] *want recognition, they want people to notice them. Yeah, that’s what I think they expect from us.”* (Participant 11, female, Indian, 20+ years of work experience)

Another participant elaborated:

*“So, under employee value proposition, people are even going further to say, what can you provide for my family? Can you provide a discount for my kids’ school? Can you provide anything more than that, in terms of my parents, you know, how do I make sure they are protected? So, the employee value proposition needs to touch on a lot of things in terms of what the guys* [employees] *actually get, others are soft expectations and others are of financial or tangible nature, which you can easily quantify.* (Participant 12, male, African, 6+ years of work experience)

#### Sub-theme: Talent recruitment and retention

The ways in which companies attract and retain talent continue to change rapidly. The COVID-19 pandemic has opened new negotiating mechanisms when talents are seeking new employment opportunities and in their current employment. Some of the comments support that adjusting to pre-COVID-19 pandemic ways will be detrimental to attracting and retaining talent, which forces companies to have to adjust their practices:

*“Competition is at a high and companies need to adjust their practices if they want to attract and retain talent. I think converting to the pre-COVID ways can be detrimental to talent.”* (Participant 9, female, coloured, 16+ years of work experience)

*“That’s what the guys* [employees] *are saying and we are saying that we need more collaboration from the office. So that is something that will be interesting as it unfolds because on the one side, you will lose good talent that will say, I prefer staying in Pretoria, I get a lot done from home and do not prefer to come to the office daily and stuck in traffic.”* (Participant 12, male, African, 6+ years of work experience)

#### Sub-theme: Quality of outputs

An interesting comment made by one of the line managers is that when employees are engaged, they will likely produce quality outputs that will contribute to the bottom line of the business, as supported by the following response:

*“…it’s just a situation and I’ve had that happen, where home life was falling apart, and work as well with poor performance. Again, it’s not poor performance. It’s poor performance because your mind is distracted and you’re completely elsewhere and I think, again, it comes back to if you say you engage with employees…”* (Participant 7, male, Indian, 6+ years of work experience)

#### Sub-theme: Meaningful work and job satisfaction

While this sub-theme occurred the least frequently in the findings, it still offered important insights. Employees expect to be provided with work that is meaningful and where they can find purpose in their work, as well as feeling that their work/job makes a difference in the business objectives which can ultimately lead to job satisfaction.

*“…how their role can actually add value and bring this purpose to life and connecting what they do to the greater purpose.”* (Participant 13, male, African, 11+ years of work experience)

*“It’s a continuous process for us to make sure that we understand, like I said, every individual has different expectations. Others could be money, others could be a career, and others could be job satisfaction.”* (Participant 12, male, African, 6+ years of work experience)

### Main theme 2: Business nature, structure, and operations

Line managers alluded that employees expect to understand the business purpose, vision and mission, as well as what their expected contributions to achieving the mission and purpose of the organisation are. The COVID-19 pandemic has changed the structure and operation of the business. Similarly, in the South African bank where the research was conducted, line managers believe that employees will expect work flexibility, giving them a sense of autonomy in their work. Teamwork is crucial in achieving the business strategic objectives and the role of the psychological contract and meeting expectations of employees, technology, and leadership are crucial to help the organisation to achieve its competitive advantage. The world of work is changing at a fast pace and requires organisations to keep abreast of the changes and adjust their practices, thus remaining sustainable.

#### Sub-theme: Business purpose and expectations

This sub-theme occurred most frequently within the broader main theme of business nature, structure, and operations. Some of the participants mentioned that it is their responsibility to reaffirm the purpose of the organisation and that of their respective departments with employees, as well as where the employees fit in. Line managers believe that employees expect them to provide clarity on the expectations from employees and their contribution thereof. Below are some of the comments supporting this:

*“It is reaffirming an individual’s purpose. Reaffirming the why, why are you here? What is the purpose of you being here? Are you here just to be paid on the 25*^th^* day of the month and that’s the tick box exercise, or are you here because you believe that we are building the best business bank in the world? Are you here because you believe that if we make a difference in an SME’s life, we* [are] *changing the lives of the employees? We’re changing the lives of people in the country and changing the economy of this country.”* (Participant 11, female, Indian, 20+ years of work experience)

*“It is the extent to which people are connected to the organisation in which they feel that they can apply discretionary effort freely and openly. It’s the connection that they have with the organisation, with the colleagues at work, with the work that they do, and having a sense of purpose in the sense of alignment between the personal values* [and] *organisational values*.” (Participant 2, female, African, 16+ years of work experience)

On the other hand, some of the line managers took ownership of how they can be intentional in driving the business purpose and leading the employees in their teams with clear guidelines and direction. Below are some of the quotes from the participants supporting the preceding statement:

*“…how do we make the people agenda that much clearer, starting with putting it on paper and making it explicit, what is it that we stand for? What is our role? What is our purpose? What is our vision? What is our mission? Why do we exist? What is the right that we have as finance to exist in this organisation, and begin to create fun and excitement and energy back?”* (Participant 13, male, African, 11+ years of work experience)

*“I think in my opinion, employee engagement is on various levels, I think you start, and you can almost cascade down. So, you can start with a contract that the employee has and their performance, the goals, performance deliverables that they have to achieve so that these are very clear guidelines, very clear communication of what is expected of them…”* (Participant 8, female, white, 20+ years of work experience)

#### Sub-theme: Work flexibility/autonomy

This was the second most frequently occurring sub-theme under the broader theme of business nature, structure and operations. Given the current global COVID-19 pandemic, managers expressed that employees’ expectations after the pandemic will be for the bank to adopt a hybrid working model as an approach to attract and retain talent. This was derived from the sub-themes that emerged from the responses of the line managers. It became clear that this expectation will be one of the expectations that employees have of their employment experience. Below are some of the verbatim quotes from the participants highlighting the foreseeable need for flexibility and work-life balance:

*“The flexibility element is quite a huge one. Yeah, so being able to operate effectively in this hybrid environment, they still prefer their working from home thing and being able to come into the office when they need to. That’s the key thing that I’m picking up, that flexibility*.” (Participant 10, female, African, 11+ years of work experience)

*“I think the psychological contract will be one of more flexibility – with staff being able to choose for themselves whether they want to work in the office or at home, and when they work.”* (Participant 6, female, white, 16+ years of work experience)

Another participant added an interesting comment regarding work flexibility. While employees may expect it, it may not be granted, as supported by the below quote:

*“They can expect it* [remote working], *but it doesn’t mean it’s going to happen…”* (Participant 8, female, white, 20+ years of work experience)

#### Sub-theme: Teamwork

Some of the participants below indicated that having an inclusive team where support is provided and trust relationships are formed, is important. Below are some of the thoughts that the participants had to say:

*“You don’t pack your emotions one side and come in and function as the machine and to set the tone like that* [socially aloof work culture] *because to be vulnerable, it means you allow the opportunity for us to trust each other, and to be open and frank discussions and it fosters an opportunity to handle conflict and just to build trust…”* (Participant 9, female, coloured, 16+ years of work experience)

*“… I think it’s about those relationships that you’ve built with individuals as well. That personal trust and rapport that you have built.”* (Participant 4, female, coloured, 11+ years of work experience)

#### Sub-theme: Characteristics of the institutional psychological contract

An institutional psychological contract, which includes open communication, trust and care, is paramount in keeping employees abreast of what is happening in the organisation and was found to be one of the employees’ expectations. It is evident that most line managers have the quality of keeping communication lines open and building trust and safe working relationships with their employees. Their employees are allowed to have freedom of speech, voicing any ideas and or concerns to take their respective departments forward. Below are some of the verbatim comments from the participants as derived from the sub-themes:

*“All you can do is really be seen to care, but also to demonstrate that you care, through checking in…”* (Participant 13, male, African, 11+ years of work experience)

*“They also expect fair and reasonable remuneration, along with consistent and appropriate feedback on their performance.”* (Participant 6, female, white, 16+ years of work experience)

*“They* [employees] *get demotivated* [when their expectations are not met]. *So, I’ve got candidates with a lot of potential, but they find themselves in a rut, you know, they don’t see the way out and then that comes through in the productivity and their work ethic.”* (Participant 1, female, white, 16+ years of work experience)

Some participants also highlighted the psychological contract/expectations on the part of the line manager, as supported by the following:

*“My first day* [with employees] *on the job is about laying the ground rules, and when laying the ground rules, you don’t read the martial law pretty much. You must let whoever you’re working with know how you work because each one of us has different work ethics…”* (Participant 3, male, African, 16+ years of work experience)

Other participants mentioned that not meeting the expectations that employees have of their employment experience will likely damage the employee’s loyalty and trust with the organisation and line managers, and that would become a bigger problem to resolve. A trusting working relationship will help the bank achieve the objectives set. Once the loyalty and trust are broken, employees will tend to withdraw and ultimately do the bare minimum without going over and above their call of duty. Below are some of the comments that were made by the participants:

*“If we break that trust, it breaks everything and you can’t piece it back together, the way it was if that trust is broken. It’s totally difficult.”* (Participant 5, female, African, 16+ years of work experience)

*“Yeah, I think any breach depending on what it is, it’s going to lead either to the following, right: number one, it is complete withdrawnness… I put nothing more, I put nothing less, I just do what I’m required to…”* (Participant 7, male, Indian, 6+ years of work experience)

On the other hand, if these expectations are not met (i.e., if the psychological contract is breached), employees will likely leave the organisation to look for a more conducive work environment elsewhere. Comments supporting this statement from the participants are included below:

*“It would very likely result in higher staff turnover, higher unplanned absenteeism, missed deadlines, lower participation levels, and, potentially, grievances or lower engagement scores on regular surveys conducted by the organisation.”* (Participant 6, female, white, 16+ years of work experience)

*“We had a situation at some point with one particular branch, the guys* [employees] *were disengaged for various reasons linked to the leader of that particular centre, you would just see the guys* [employees] *putting their headset as they walk in, they put their headset because, they don’t want to talk, they just engage with the work that they need to do and that tends to create a situation where people resign and leave.”* (Participant 12, male, African, 6+ years of work experience)

#### Sub-theme: The role of technology

The COVID-19 pandemic has accelerated the use of technology and forced businesses to adopt the 4IR concepts/way of work by using digital tools to remain able to effectively service their clientele. During the pandemic, companies have had to introduce electronic tools to serve their clients and like many other businesses, the bank where the study was conducted had to introduce the same. Electronic tools and platforms such as Microsoft Teams, Skype, Zoom etc., will continue to be much-needed modes of communication, improving processes and helping the organisation to reach a wider range of clientele, ultimately providing employees with the flexibility of working from anywhere.

*“I think the use of technology to collaborate* [is one of the key employee expectations], [as well as] a*utomation to help improve the time spent on tasks and improve processes.”* (Participant 13, male, African, 11+ years of work experience)

However, another participant highlighted a challenge associated with the use of technology due to the COVID-19 pandemic, as supported by the following quote:

*“So, the bigger challenge we’re gonna be faced with going forward is, we become more antisocial, due to COVID. We* [are] *already becoming antisocial, due to technology. One can say we were still social, but distance relationships in terms of digital that we started adopting, and how do we then balance what COVID has forced us to do, working digitally, adopting the digital tools, as well as how do we balance the social life in a different context?”* (Participant 12, male, African, 6+ years of work experience)

On the other hand, companies will need to implement tighter security measures on the devices that are provided to their workforce to protect company information. Below is a comment from one of the participants supporting this:

*“New risks might also emerge as a result of working from home. Banks need to consider putting more security in the electronic devices that we use.”* (Participant 3, male, African, 16+ years of work experience)

#### Sub-theme: Leadership

Line managers mentioned that employees expect consistent and appropriate feedback, support and guidance from organisational leadership for their growth and development. Below are some of the comments from the participants supporting this:

*“…I think from my perspective with my team, we* [are] *comfortable to have weekly or bi-weekly check-ins. Weekly check-ins concerning whether it be work performance or work deliverables, how we can help each other to remove any bottlenecks, as well as your self-development. If there is a challenge, I would like to believe they’re open to letting me know*, [and if] *there is a challenge, either I contribute to the challenge, or there is a challenge too big for them and they need my help or your support, or there is a sticky situation, and they need your* [my] *direction.”* (Participant 9, female, coloured, 16+ years of work experience)

*“You’re not able to promise someone a promotion because, for a promotion to be available, there must be a vacancy… So, what can you do as a line manager? You can provide support, you can provide some sort of career guidance in terms of where to look*, [or] *how to look for it. Advise in terms of getting a mentor, and assisting them, getting someone who can help them if you* [I] *can’t help them because I can’t help them all the time.”* (Participant 3, male, African, 16+ years of work experience)

#### Sub-theme: Organisational culture and change management

While this sub-theme emerged the least frequently, it still presented important insights. The bank is currently going through a merger with another bank, which means that two cultures co-exist, and the change management process has to be effectively implemented to remove any potential fears from employees. However, it is important to mention that the bank has seen staff turnover increase as a result of the merger. Below are some of the initiatives of line managers to help their employees through the change and confirm the staff turnover:

*“…the bigger piece that I’m currently doing is the culture change. The purpose of that is obviously to empower from within so that you don’t have to hire from external, as well as making sure that there’s an integration that these guys* [employees] *understand what the parent expects”* (Participant 12, male, African, 6+ years of work experience)

*“When we went through the buyout with XXX bank, there was a lot of fear, because of the unknown. Nobody knew what to expect, which created fear in the current market regarding employment, you had a lot of guys jumping ship.”* (Participant 7, male, Indian, 6+ years of work experience)

### Main theme 3: Nature of work environment

Line managers believe that employees expect their employers to provide them with environmental occupational health and safety, to be treated with respect, fairness and transparency, and ultimately to be provided with job security.

#### Sub-theme: Occupational health and safety

This sub-theme was the most occurring sub-theme within the broader main theme of the nature of the work environment. It is important to mention again that the COVID-19 pandemic has changed the lives of employees forever. Employees now expect more safety in the workplace to provide them with comfort that their employer cares about their health and safety by ensuring that protocols such as those for COVID-19 are strictly enforced. The below quotes provide a glimpse into the health and safety expectations that employees have:

*“From a COVID perspective, following the relevant regulations and protocols, reinforcing that as an organisation* [is important]*.”* (Participant 3, male, African, 16+ years of work experience)

*“Organisations* [need] *to be a lot more aware of employee safety and mental well-being has become something that previously, was on the periphery, and now it’s really at the centre of organisational life where organisations are expected to play a more active role in helping employees manage mental health issues. So those are the things that I think will shift.”* (Participant 2, female, African, 16+ years of work experience)

#### Sub-theme: Respect, fairness and transparency

Employees’ expectations should be treated with respect, fairness and transparency. Below are some of the comments from line managers indicating this:

*“Employees in my team expect to be treated with respect, fairness, and transparency. They also expect fair and reasonable remuneration, along with consistent and appropriate feedback on their performance.”* (Participant 6, female, white, 16+ years of experience)

*“I think it’s the basic things, its fair pay, fair working conditions, the support for the development, and just general support for the humanity.”* (Participant 2, female, African, 16+ years of work experience)

#### Sub-theme: Job security

While this sub-theme showed the least frequency of occurence in the findings, it still offered important insights. Job security is important to employees, and they expect it from their employment experience. Again, given the unstable economic conditions that have been perpetuated by the pandemic and merger that the bank is undergoing, employees expect their jobs to be secure so that they can fend for their families and continue to have a good quality of life. Below are some of the comments from the participants:

*“I think job security is important for them.”* (Participant 8, female, white, 20+ years of work experience)

*“…so, with that comes people feeling threatened about their job, are we still going to have a job, these guys are taking over everything that we’ve built for the past 60, 40 years.”* (Participant 12, male, African, 6+ years of work experience)

### Main theme 4: Emotional needs

The COVID-19 pandemic has heightened the emotional needs of employees and the line managers confirm and pledge their support to those employees who may have been affected by either losing a loved one or being psychologically impacted. The bank has Employee Assistance Programmes (EAP) to help employees with professional counselling.

#### Sub-theme: Humanity/sense of belonging

This sub-theme occurred most frequently within the broader main theme of emotional needs. Employees expect to have a sense of belonging from their employment experience, to be treated as individuals and to be cared for. Below are some of the comments from the participants:

*“…we must never take away that human element. COVID has brought* [us] *back to say, don’t forget about the person behind the scenes, and I think it’s allowed me to personally think a little bit differently about how I engage and it’s not just go, go, go, but to stop, put the brakes on, assess the individual.”* (Participant 11, female, Indian, 20+ years of work experience)

*“The bigger part is spending more time on things that will have a direct impact on people’s lives in terms of them being able to succeed. So, we need to go back to the basics and see how we can invest in people and support them.”* (Participant 12, male, African, 6+ years of work experience)

#### Sub-theme: Emotional support and morale

According to line managers, employees expect to feel that they are being provided with emotional support and in turn, their morale will increase.

*“On the negative side, it has been difficult to engage personally with people and to understand their body language and their responses. It has also been difficult, without being able to “see” the people, to know when they are burning out or suffering.”* (Participant 6, female, white, 16+ years of work experience)

*“Well, it’s around engaging with them. You know, having conversations with them. You can see when a person is present*, [or] *not present, it’s not about always talking about work, you know, doing that personal check-in with the individual just finding out how they’re doing …”* (Participant 4, female, coloured, 11+ years of work experience)

#### Sub-theme: Loss

While this sub-theme showed the least frequent occurrence in the findings, it still revealed important insights. Many employees have either directly or indirectly suffered losses. The COVID-19 pandemic has had a severe negative impact on the emotional and psychological needs of employees. Emotional and psychological support continues to be a dire need for employees. Below are some of the verbatim comments from line managers that support the sense of loss.

*…a staff member in another team was struggling with side effects from her second vaccine while grieving the loss of a loved one to COVID-19…”* (Participant 6, female, white, 16+ years of work experience)

*“…it was difficult for people because you were dealing with loss, and you just had to continue with life. You know, it was just the people didn’t have the time to go through the bereavement cycle.”* (Participant 4, female, coloured, 11+ years of work experience)

## Discussion

According to Argyris (1960), employees expect their managers to develop, grow and use their initiatives, in doing so respecting the right of the organisation to evolve in return. This study supports the literature as it found that line managers expect that employees want managerial support, empowerment and career growth, fair and consistent feedback on performance, fair and reasonable remuneration and employee benefits, and meaningful work and job satisfaction. It further found that if employees do not feel valued by the organisation and or the work environment is not conducive, they will likely look for opportunities elsewhere or withdraw from the purpose, which will ultimately result in lowered performance. This is supported by the existing literature, as well as the cognitive dissonance theory outlined earlier in this study, which states that a breach of the psychological contract can lead to either party feeling psychologically and emotionally aggrieved, leading to a loss of loyalty, motivation, and overall duty underperformance (Festinger, 1957; Alcover et al., 2017; Abbas and Al Hasnawia, 2020; Saurombe and Barkhuizen, 2022; Swanepoel and Saurombe, 2022). Tomprou and Lee (2022) also indicate that psychological contracts are important to organisations because managing the expectations of employees during the induction stage has a direct influence on the performance of the organisation by the employees and their commitment to the organisation. Further, if employees believe the organisation has not kept their expectations of the psychological contract, they may neglect some of their duties and underperform, while those who have highly marketable skills may leave the company (Tomprou and Lee, 2022).

The research also found that employees expect to be remunerated accordingly and to be provided with meaningful work. This creates career growth opportunities within the organisation. Similarly, the literature highlights that the transactional psychological contract elicits insignificant commitment from the involved parties as the emphasis is placed on short-term financial requirements (Rousseau et al., 2018). In other words, the transactional psychological contract focuses on specific, short-term, and financial requirements (Rousseau et al., 2018). Liu et al. (2020) further add that it is important for employers to mutually understand the obligations and the employees’ perception of the fulfilment of the transactional psychological contract. Additionally, clarity on strategic objectives leads to increasingly constructive effects on employees’ psychological contracts and unambiguous messages. Unclear strategic objectives lead to the opposite, increasing the odds of adverse effects (Baruch and Rousseau, 2019). According to the study conducted by Braganza et al. (2021), there is a positive relationship between work engagement and trust. A trust relationship is essentially at the core of an employment relationship (psychological contract). It influences the behaviours of the employer and employer. Trust also helps to prevent psychological contract breach from either the employer or employee. According to the findings, when the teams understand their purpose and clear expectations, trust relationships are formed, and they are willing to support each other to achieve the teams’ objectives, which in turn feeds into the organisation’s strategic objectives.

In support of the existing literature and unlike transactional psychological contracts, relational psychological contracts are based on broad and long-term responsibilities and social emotions, such as dedication and loyalty (Chan, 2021). This supports the findings of the research. Participants referred to trust relationships and team support as very important as represented by the theme of teamwork. This study found the following sub-themes that have direct links to employee engagement: managerial support, empowerment, and career growth; performance management; quality of outputs; business purpose and expectations; and characteristics of the institutional psychological contract. According to what was found in the existing literature, employee engagement entails connecting with colleagues and managers in a meaningful way (Naidoo et al., 2019; Jarrar, 2022).

Managers also believe that employees expect to be stretched to achieve beyond the scope of their jobs. This helps them to grow in the career direction that they choose while broadening their knowledge to excel in the current job, as found in the existing literature (Deas and Coetzee, 2021). Similarly, this study showed that line managers believe that employees expect a supportive work environment where they feel empowered to take charge in the execution of their jobs while remaining within guidelines that they (line managers) provide. Career growth was another emergent sub-theme as line managers believe that employees expect a learning environment where employees can grow in their careers through the learning opportunities and earn more (Magagula et al., 2020; Saurombe and Barkhuizen, 2020). Performance management was the second-highest sub-theme in the broader main talent management theme. Employees contribute positively to organisational effectiveness and objectives when they feel that their managers are interested in their development (Naidoo et al., 2019). Similarly, this study uncovered that most line managers agreed that this is a major component when it comes to leading their teams and contracting and communicating performance targets and expectations to employees, thus ensuring alignment between strategic objectives and employees’ performance goals. Performance management also helps line managers to measure the quality of outputs in line with the contracted performance goals to identify whether employees are achieving the contracted performance goals and beyond.

Additionally, performance management is also a mechanism that line managers use to communicate the business and employee’s goals and expectations in this regard to ensure that employees understand how their unique contributions can add value to the bigger business objectives (Lambert et al., 2020). According to Son and Kim (2021), employee engagement is when employees are connected to the organisation and its mission and vision. Similarly, this research uncovered that employee engagement is the extent to which employees are connected to the organisation so that they can freely and openly apply their discretionary efforts. It is also the connection that employees have with the organisation, colleagues, the work they do, and the extent of their sense of purpose or how it aligns with their personal and organisational values. Men et al. (2021) found that leadership communication fostered employee trust through the motivating language theory during the COVID-19 pandemic. An institutional psychological contract such as open communication, trust and care are essential components of employee engagement. Similarly, it prevailed in this study that line managers embody these components in ensuring that their employees are engaged and cared for using communication, thus enhancing the trust relationship.

The literature reveals that employee engagement refers to the extent to which an employee’s mind and heart are captured by an organisation, helping organisations to perform better (Agarwal and Sajid, 2017; Naidoo et al., 2019). Energy, involvement, and positive interaction are regarded as employee engagement characteristics (Naidoo et al., 2019). Literature shows employees to be the main resources or stakeholders who enable organisations to achieve their competitive advantage and objectives (Fauzi et al., 2021) and according to Agarwal et al. (2021), employee engagement has the power to drive the performance of the organisation. Agarwal et al. (2021) further add that engaged employees understand their position and purpose in the organisation. This allows them to become motivated and driven enough to perform the tasks allocated to them with passion. Fauzi et al. (2021) identifies these employees as the engine of the organisation. Similarly, this research found that employees expect to be performance contracted, thus setting the scene on the clear expectation of the purpose, vision and strategic objectives. Business purpose is when employees understand their unique contribution and the overall strategic objectives of the organisation (Agarwal et al., 2021). Leaders are vital in providing clear guidelines and expectations in an employment relationship (Heinzel and Liese, 2021; Shingenge and Saurombe, 2022). Performance management helps to set the scene for the expectations from the start of the employment relationship (Sim et al., 2018). Thus, the aim of this study to explore the perceptions of line managers about how obligations and expectations of employees and employers in the form of the psychological contract can influence employee engagement in a South African bank was achieved.

The impact of the integration of technologies of the 4IR on the workplace in the banking sector was also explored. Not only does the banking sector have to adapt to the 4*^th^* Industrial Revolution, it also has to deal with the impact of the COVID-19 pandemic, which has caused an economic downturn across the globe (Wu and Olson, 2020). Most bank assets are in a form of loans such as house mortgages, personal and business loans, as well as vehicle instalments, and millions of individuals were forced to miss their monthly payments to banks due to numerous businesses closing (Rutkowska-Tomaszewska et al., 2021; The Banking Association of South Africa, 2021). The knock-on effect of the pandemic has negatively affected the banking industry and its employees as it has led to the loss of income for the institution and subsequent retrenchments (Wu and Olson, 2020). With more and more employees working remotely from the comfort of their homes, upholding the psychological contract has become more important than ever during this period to ensure that employees meet organisational targets on time (Mmamel et al., 2021).

As the study was done during the COVID-19 pandemic, some findings related to the acceleration of technology use due to the pandemic also emerged. According to Lund et al. (2021), three broad trends have been accelerated by and are likely to recede after the COVID-19 pandemic. The three trends in question are remote work, digitalisation, and automation. According to the report, remote work after the pandemic will depend on how companies are able to adjust and introduce new worker flexibility models while not completely discontinuing in-person key activities for greater effectiveness. Businesses’ potential to accelerate automation will depend on whether they are willing or able to invest in technologies that will enable them to reconfigure work to capture other opportunities after the pandemic. The report further highlighted some of the benefits that are associated with the remote working model, being greater flexibility for employees and business efficiencies. This research uncovered that the expectation after the pandemic will be greater flexibility, which has also been mentioned as one of the benefits of remote work in the report by the McKinsey Global Institute (Alexander et al., 2020). However, companies will have to keep ensuring that the place of work (office) continues to be safe from a COVID-19 perspective, thus providing comfort to employees who are expected to go to the office for office engagements or as part of a hybrid approach. Technology integration and tighter security measures as a result of automation also emerged. When employees work remotely, they are more exposed to technological risks. Companies will have to mitigate these risks to protect company information. Employees will continue to have emotional needs and a need for support as they recover from the losses that were brought by the pandemic. Talent attraction and retention themes also emerged, and companies will have to adjust their practices to open themselves up to a wider pool of talent that can help to take the company forward.

The bank where the study was conducted is undergoing a merger and while some of the employees are excited about the merger, some of the employees have been affected by the changes, making job security an emergent theme. The psychological contract is important in managing organisational change during external/internal environment changes such as COVID-19 or a merger (Smith, 2021). It is significant for embedding an organisational culture in employees (Sandhu and Sharma, 2022). The company is making strides in ensuring that their employees remain protected and secure in their jobs and that effective change management strategies to ensure a smooth integration are implemented, as was also found to be critical in the study by Saurombe and Barkhuizen (2022).

## Conclusion

This study aimed to explore how the psychological contract influences employee engagement in a South African bank. The study uncovered several similarities between the literature and the findings of this study on what employees expect from their employment relationship from a psychological contract perspective. Line managers believed that employees in the first instance expect talent management strategies such as managerial support, performance management, career growth, benefits, etc., to be in place. Second, understanding the nature of the business, its structure, and the goals of the business and the role of the individual are important.

Additionally, the study highlighted the constantly changing expectations in the rapidly changing world of work, particularly in the context of the COVID-19 pandemic. The COVID-19 pandemic is changing the nature of work and employees’ expectations. Employees’ expectations that emerged from the study as a result of the COVID-19 pandemic include flexibility and increased empowerment, teamwork, the role of the psychological contract and technology, as well as visionary and progressive leadership (Islam et al., 2021). The COVID-19 pandemic also changed expectations of the work environment as line managers perceive an increased need for occupational health and safety, respect, fairness, and transparency, as well as job security as a result of the turbulent and uncertain times brought by the COVID-19 pandemic. It was evident from the findings of the study that the COVID-19 pandemic has influenced how quickly organisations need to adapt and adjust their talent attraction and retention mechanisms. It is paramount that employers and line managers are fully equipped to meet those expectations.

This study contributes to and shapes the theoretical knowledge and research that has been done on the psychological contract within the banking sector in South Africa. The findings of this study are instrumental in equipping and helping current and future line managers to improve their working relationship with employees and highlight the need for the HR function to timeously adapt to the constantly changing world of work as influenced by the 4IR and technology. This study may further enable and empower line managers to openly have conversations to understand what the expectations of their employees are, and actively work on meeting those expectations. This study may also create awareness in companies and among future line managers of the importance of managing and honouring expectations, not only in the banking sector within South Africa, since employee engagement is critical to the success of any organisation, regardless of sector or country. The findings uncovered in this study can also be useful to policymakers at South African financial institutions to identify improvements and shape the future of employment policies.

The study had its limitations. A qualitative research approach was used for this study, which means that the research missed out on the strengths of alternative approaches. The study focused only on line managers’ perspectives on the influence of the psychological contract on employee engagements and did not capture a mixed audience (i.e., employees’ perspectives). The study was conducted in the Gauteng region, which limits the generalisability to other populations. Since the purposive sampling strategy was used in this study, the study did not benefit from other sampling strategies and generalisability was further reduced. The study focused on line managers employed by only one and no other South African bank, so the study is not representative of other banks in South Africa. Furthermore, the physical dimension of employee engagement was not explored, as the study was conducted during the lockdown due to the COVID-19 pandemic.

The limitations discussed above inform various recommendations for future research. The influence of the psychological contract on employee engagement in a South African bank by focusing on exploring the employment expectations of employees and comparing those to line managers’ perceptions is recommended. Furthermore, future research could expand the geographic location of participants to improve the generalisability of the findings. Exploring other banks operating in South Africa and comparing the findings between the various banks could also be of value. Employing a quantitative research approach to curtail some of the limitations presented by the qualitative research approach is also recommended.

The study recommends that organisational policies include some of the possible unwritten expectations to improve talent attraction and retention. Financial institutions in the private sector should consider adopting a flexible/hybrid work model as part of their talent attraction and retention strategy. Considering the current context of the global pandemic, the 4IR and the effects that it had on employees, organisations in the financial services sector should consider making it part of their policy for employees to have an opportunity to work from home, as the findings suggest that employees would prefer working from home or at least having a hybrid working model in place.

It can therefore be concluded that line managers perceive that the psychological contract influences employee engagement in the South African banking workplace, irrespective of the changes in psychological contract caused by 4IR and the COVID-19 pandemic.

## Data availability statement

The raw data supporting the conclusions of this article will be made available by the authors, without undue reservation.

## Ethics statement

The studies involving human participants were reviewed and approved by University of Johannesburg, College of Business and Economics, Department of Industrial Psychology and People Management Research Ethics Committee. The patients/participants provided their written informed consent to participate in this study.

## Author contributions

This article was adapted from the master’s dissertation of DN, who executed the research, while MS and RJ were the study leaders and provided conceptualization guidelines, assistance with data analysis and editorial inputs. All authors contributed to the article and approved the submitted version.
